# Night Air

**Published:** 1860-09

**Authors:** 


### NIGHT AIR.

"An extraordinary fallacy is the dread of night air. What air can we breathe at night but night air? The choice is between pure night air from without, and foul night air from within. Most prefer the latter. An unaccountable choice. What will they say, if it is proved to be true that fully one half of all the disease we suffer from is occasioned by people sleeping with their windows shut? An open window most nights in the year can never hurt any one. This is not to say that light is not necessary for recovery. In great cities, night air is often the best and purest air to be had in the twenty-four hours. I could better understand shutting the windows in towns, during the day, than during the night, for the sake of the sick. The absence of smoke, the quiet, all tend to make night the best time for airing the patient. One of our highest medical authorities on consumption and climate, has told me that the air in London is never so good as after ten o'clock at night. Always air your room, then, from the outside air, if possible. Windows are made to open, doors are made to shut—the truth which seems extremely difficult of apprehension. Every room must be aired from without—every passage from within."

Many a man will wake up on the other side of the "Styx" and as soon as he rubs his eyes a little, will exclaim, that angel of a woman, Florence Nightingale, has killed me. We protest against any body making publications on human health, except educated physicians, and we counsel those of our readers who wish to be on the safe side, to give a wide berth to all rules and regulations and suggestions and dicta about the preservation of health and the cure of diseases, unless they bear the name of some medical man of eminence, or of some medical publication of acknowledged authority. Miss Nightingale is a professional nurse, a woman of superior powers of observation, comparison and reflection, and has written a most admirable book on "Nursing," but not having had a previous education in the great general principles of anatomy, physiology and hygiene, she has, in the extract which heads this article, left an impression on the mind of the general reader, which is at once confused, erroneous and of vital importance. There is important truth running through the whole of it, but for want of discrimination and definiteness it may be productive of more injury than good. We are in the habit of saying to consumptive persons, "Any air is better for you, than in-door air;" but then we add the proviso that the hours, including sunrise and sunset, should either be spent in the house or should, if they choose to go out of doors, find the stomach fortified with breakfast or supper, for the reason that in warm weather in all flat, luxuriant localities a deadly air, called miasm, which is an emanation from the decomposition of vegetable matter, where there is warmth and moisture, hovers over the surface of the earth, within its first ten feet, and if this air is breathed into the lungs, especially when the stomach is empty, disease, more or less speedy, and more or less intense, will be inevitably excited, from a slight intermittent, to continue for days, weeks or months, to a malignant, or putrid, or ship fever to cause death in a few hours. On the general principle that a heat of eighty degrees and upwards, rarefies miasm, and carries it beyond our reach, while a temperature under eighty of Fahrenheit, brings it to the surface, where it is breathed, and its poison introduced into the system, a few practical rules may be deduced, which are of almost universal application and are of incalculable importance.

*First.* In cold weather, out-door air, day or night, is purer than any within doors possibly can be.

*Second.* In warm weather, the air including the hour of sunrise and sunset is pernicious to all, sick or well—especially to the young, the old and the feeble.

*Third.* In warm weather, especially in the fall of the year, when fall sicknesses prevail, persons who sleep on the ground-floor should keep all outer doors and windows shut, and inner ones open.

*Fourth.* In cities as well as in the country, those who sleep in the second or upper stories may safely open their windows at bed-time, for by that time the miasm has been carried by the cold to the surface of the earth; but summer and winter, the doors of upper chambers, opening into the halls should be closed, so as to keep out all bad airs from below, whether the miasm arising from decaying vegetables, or the malaria, the "mal," the "bad" air from kitchens, dining-rooms, cellars and the like.

Upper rooms may be safely aired during any portion of the twenty-four hours, and should be aired from without. Lower rooms should be aired in the heat of the day, and closed while slept in at night, only opening the inner doors.

The "extraordinary fallacy" is in the fair lady herself, conveyed in the two inquiries at the commencement of the article. The air breathed in a lower chamber, with doors and windows closed early in the evening, is not the night air, it is the day air, only mixed with night air, miasm coming through small crevices, and so rapidly rarefied by the greater warmth of the room, of some ten degrees perhaps, that it becomes in a measure innocuous. The choice is not between pure night air from without, and foul night air from within, it is between concentrated cool miasm from without, and a warm day air with comparatively little miasm from within. It is incomparably better to sleep in poor warm indoor air, than in an outdoor air which is saturated with miasm. Besides, we have met with a number of persons, men and women, who never failed to be injured, to be made more or less ill the very next day by sleeping with open windows winter or summer, however well protected from draughts; so that, however good the general rule, to have a window or door open in a chamber for sleeping, persons who find by actual observation that it is injurious to them, should not persist in fighting against the peculiarity of their system, against their "Idiosyncrasy." Upon the whole, we think, that ladies generally had better get good husbands, make babies, happify homes, and let prosing, poetizing, and lecturing alone, for a woman is most of a queen and nearest an angel at the head of a well-ordered family of children, servants, husband, and friends.

